# Supplementary material for: Activation of the Integrated Stress Response and ER Stress Protect from Fluorizoline-Induced Apoptosis in HEK293T and U2OS Cell Lines
Source: Int J Mol Sci. 2021 Jun 6;22(11):6117. doi: 10.3390/ijms22116117 (PMC8201103; doi:10.3390/ijms22116117)
Supplement: Supplementary file 1 [file ijms-22-06117-s001.zip › ijms-1190265-supplementary.pdf]

Supplementary Figures

Activation of the Integrated Stress Response and ER Stress Protect from Fluorizoline-Induced Apoptosis in HEK293T and U2OS Cell Lines

José Saura-Esteller [1], Ismael Sánchez-Vera [1], Sonia Núñez-Vázquez [1], Ana M. Cosialls [1], Pau Gama-Pérez [1], Gauri Bhosale [2], Lorena Mendive-Tapia [3], Rodolfo Lavilla [3], Gabriel Pons [1], Pablo M. Garcia-Roves [1], Michael R. Duchen [2], Daniel Iglesias-Serret [1,4,†] and Joan Gil [1,*,†]

[1] Departament de Ciències Fisiològiques, Facultat de Medicina i Ciències de la Salut, Universitat de Barcelona-IDIBELL (Institut d'Investigació Biomèdica de Bellvitge), L'Hospitalet de Llobregat, 08907 Barcelona, Spain; jsauraes@ub.edu (J.S.-E.); isanchezvera@ub.edu (I.S.-V.); nunezvazquez.sonia@gmail.com (S.N.-V.); anamcosialls@ub.edu (A.M.C.); p.gamaperez@gmail.com (P.G.-P.); gpons@ub.edu (G.P.); pgarciaroves@ub.edu (P.M.G.-R.); daniel.iglesias@umedicina.cat (D.I.-S.)

[2] Department of Cell and Developmental Biology, University College London, London WC1E6BT, UK; g.bhosale.12@ucl.ac.uk (G.B.); m.duchen@ucl.ac.uk (M.R.D.)

[3] Laboratory of Medical Chemistry, Faculty of Pharmacy and Food Sciences and Institute of Biomedicine (IBUB), University of Barcelona, 08028 Barcelona, Spain; lorena.mendive@gmail.com (L.M.-T.); rlavilla@ub.edu (R.L.)

[4] Facultat de Medicina, Universitat de Vic-Universitat Central de Catalunya (UVic- UCC), Vic, 08500 Barcelona, Spain

* Correspondence: jgil@ub.edu; Tel.: +34-934-029-095

† These authors share senior authorship.

Figure S1

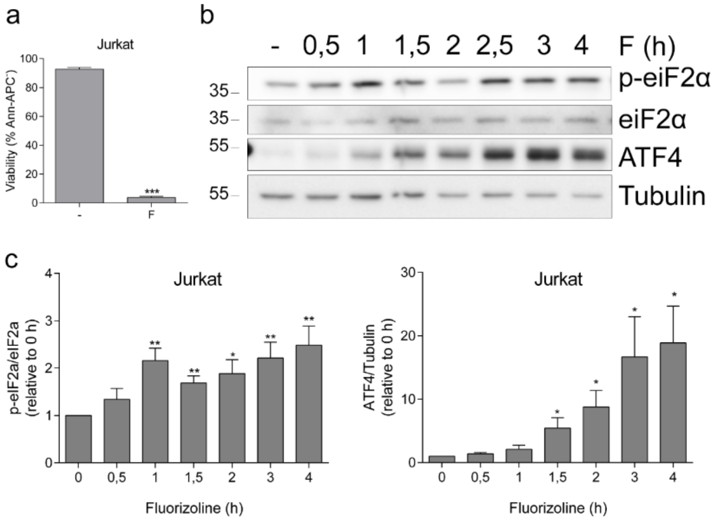

The ISR is induced after fluorizoline treatment in Jurkat cells. Jurkat cells were treated with 10 μM fluorizoline for (**a**) 24 h or (**b**,**c**) the indicated times. (**a**) Viability was measured by flow cytometry, and it is expressed as the mean ± SEM of the percentage of non-apoptotic cells (annexin V-negative). (**b**,**c**)Protein levels were analyzed by Western blot. Tubulin was used as a loading control. These are representative images of at least three independent experiments. (**c**) Quantification of relative p-eIF2α/eIF2α and ATF4/Tubulin band intensity. * p < 0.05, ** p < 0.01, *** p < 0.001. Each condition vs.-(n = 3).

Figure S2

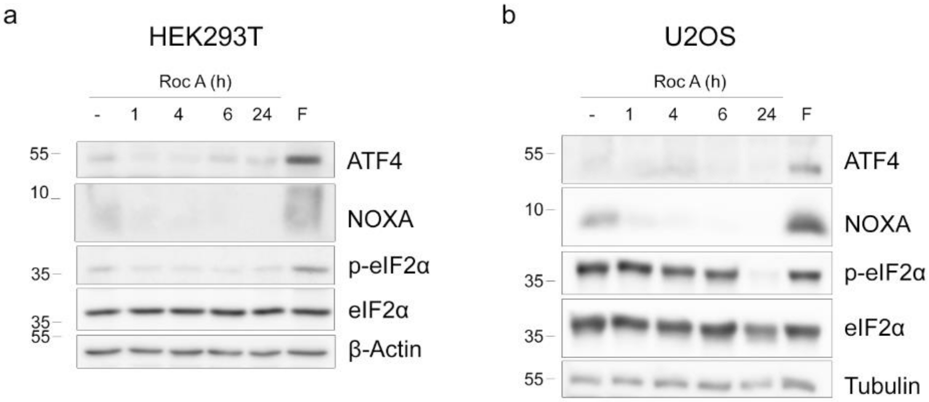

Rocaglamide A does not induce the ISR in HEK293T and U2OS cells. (**a**) HEK293T and (**b**) U2OS cells were treated with 1 μM rocaglamide A (Roc A) for the indicated times or 15 and 20 μM fluorizoline, respectively, for 6 h. Protein levels were analyzed by Western blot. β-Actin and Tubulin were used as loading controls. These are representative images of at least three independent experiments.

Figure S3

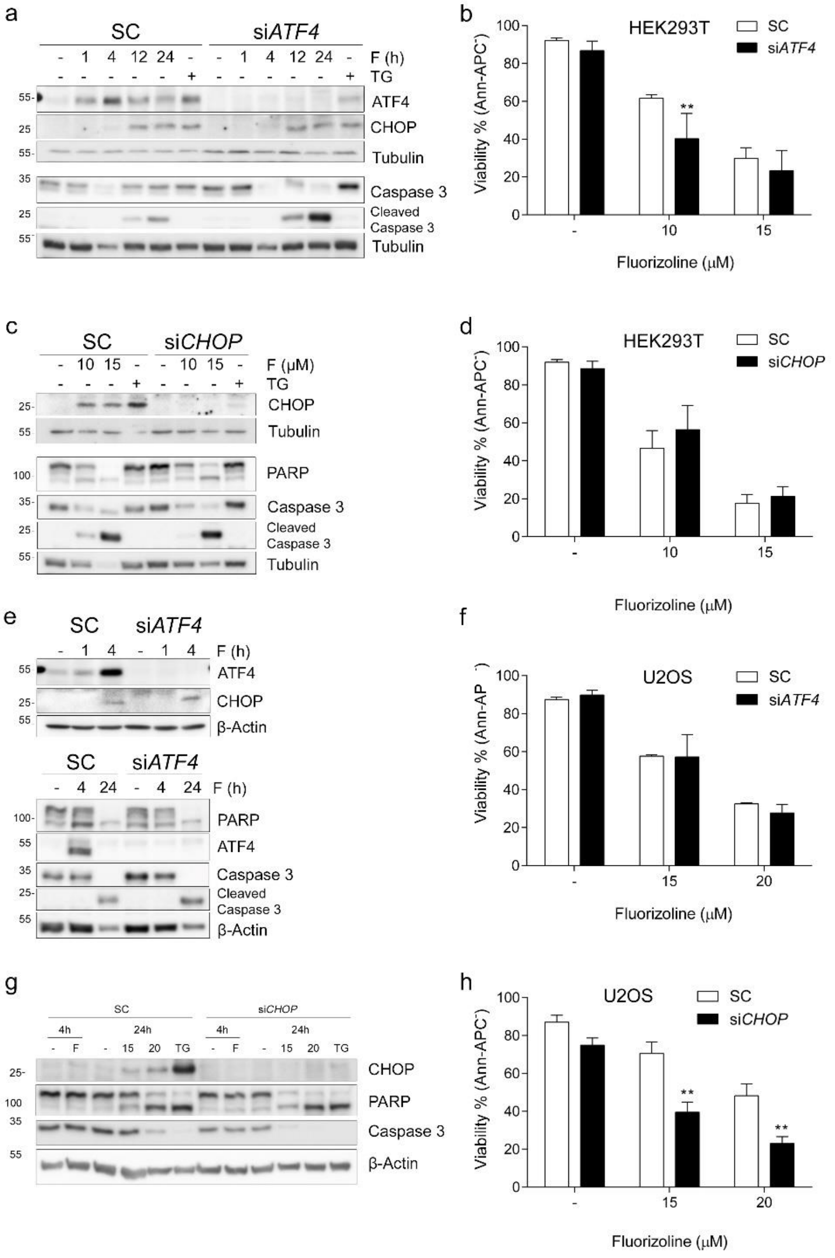

Downregulation of ATF4 and CHOP sensitize HEK293T and U2OS respectively to fluorizoline-induced apoptosis. a-d HEK293T and e-h U2OS cells were transfected with scramble (SC) or with (**a**,**b**), (**e**,**f**) *ATF4* (si*ATF4*) or (**c**,**d**), (**g**,**h**) *CHOP* (si*CHOP*) siRNA for 48 h and then treated either with 15 or 20 μM fluorizoline, respectively, with the indicated doses of fluorizoline for 24 h, or with 20 μM thapsigargin (TG) for the indicated time. (**a**,**c**,**e**,**g**) Protein levels were analyzed by Western blot. β-actin and Tubulin were used as loading controls. These are representative images of at least three independent experiments. (**b**,**d**,**f**,**h**) Viability was measured by flow cytometry, and it is expressed as the mean ± SEM of the percentage of non-apoptotic cells (annexin V-negative). * $p < 0.05$, ** $p < 0.01$. Each condition vs. SC (**b** $n \geq 4$; **d** $n = 4$; **f** $n = 2$; **h** $n = 4$).

Figure S4

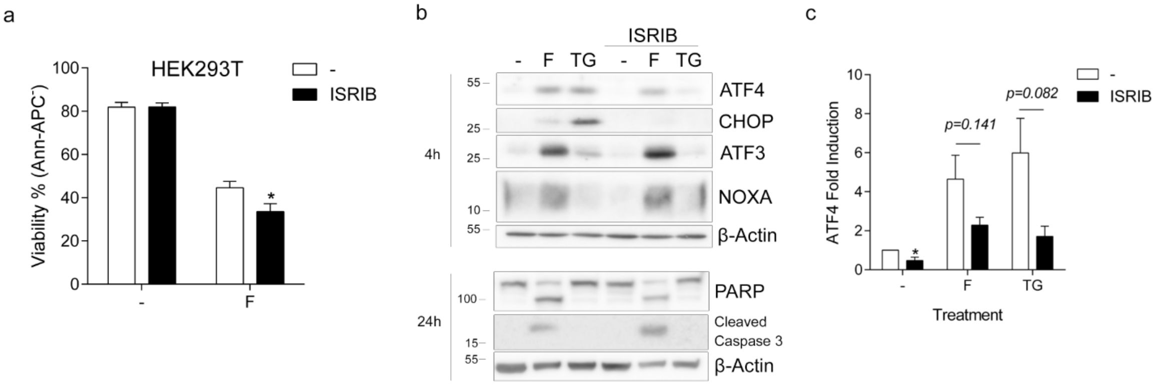

ISRIB reduces the activation of the ISR and sensitizes to apoptosis induced by fluorizoline. (**a**–**c**) HEK293T cells were pre-treated with 10 µM ISRIB for 1 h and then treated with 15 µM fluorizoline (F) or with 20 µM thapsigargin (TG) for (**a**) 24 h or (**b**) the indicated times. (**b**) Protein levels were analyzed by Western blot. β-actin was used as a loading control. (**c**) Quantification of relative ATF4/β-Actin band intensity. These are representative images of at least three independent experiments. (**a**) Viability was measured by flow cytometry, and it is expressed as the mean ± SEM of the percentage of non-apoptotic cells (annexin V-negative). * $p < 0.05$. ISRIB vs. untreated cells (−) ($n = 3$).

Figure S5

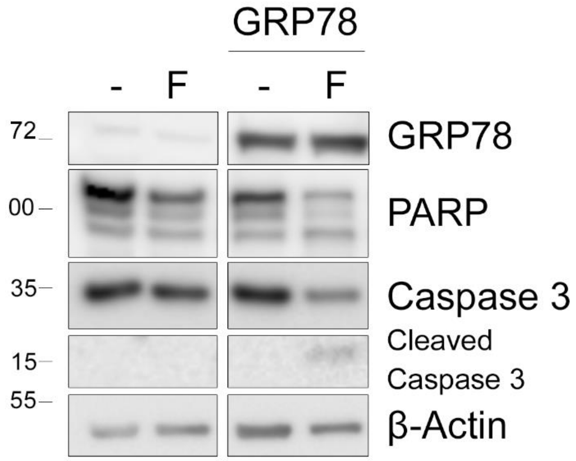

Overexpression of GRP78 increases the cleavage of PARP and caspase 3 after fluorizoline treatment in HEK293T cells. HEK293T cells were transfected with empty pcDNA or *GRP78* for 48 h and then treated with 10 µM fluorizoline (F) for 24 h. Protein levels were analyzed by Western blot. β-actin was used as a loading control. These are representative images of at least three independent experiments.

Figure S6

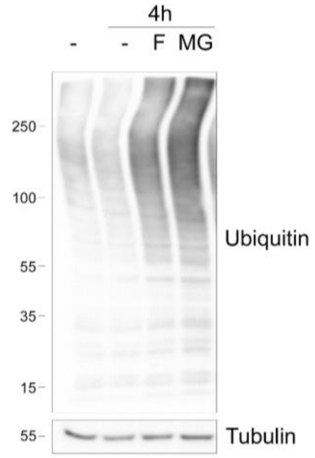

Fluorizoline causes the accumulation of poly-ubiquitinated proteins. Jurkat cells were treated with 10 μM fluorizoline (F) or 1 μM MG132 (MG) for 4 h. Protein levels were analyzed by Western blot. Tubulin was used as a loading control. This is a representative image of at least three independent experiments.

Figure S7

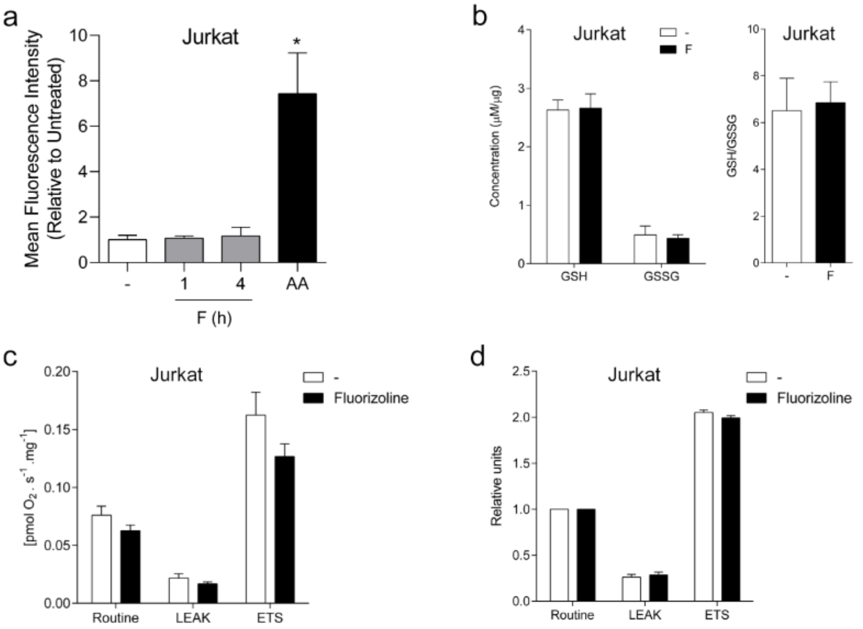

Fluorizoline does not alter ROS production, GSH concentration, or mitochondrial respiration in Jurkat cells. (**a**) Jurkat cells were treated with 10 μM fluorizoline (F) for the indicated time or 100 μM antimycin A (AA) for 1 h. Cells were loaded with 5 μM MitoSOX 15 min prior to the measurement. Superoxide production was measured by flow cytometry and is expressed as the percentage of MitoSOX positive cells. (**b**) Jurkat cells were treated with 10 μM fluorizoline (F) for 4 h. GSH, GSSG, and GSH/GSSG ratios were measured using o-phthalaldehyde. (**c**,**d**) Jurkat cells were treated with 10 μM fluorizoline for 4 h. Oxygen consumption was measured in basal conditions (Routine) after the addition of 1 μM antimycin A (LEAK) and after titration with 0.5 μM CCCP (ETS). Data are represented as (**c**) raw oxygen consumption rate and (**d**) oxygen consumption relative to Routine. * $p < 0.05$, AA vs.-(**a** $n \geq 3$; **b** $n \geq 6$; **c** $n = 4$).

Figure S8

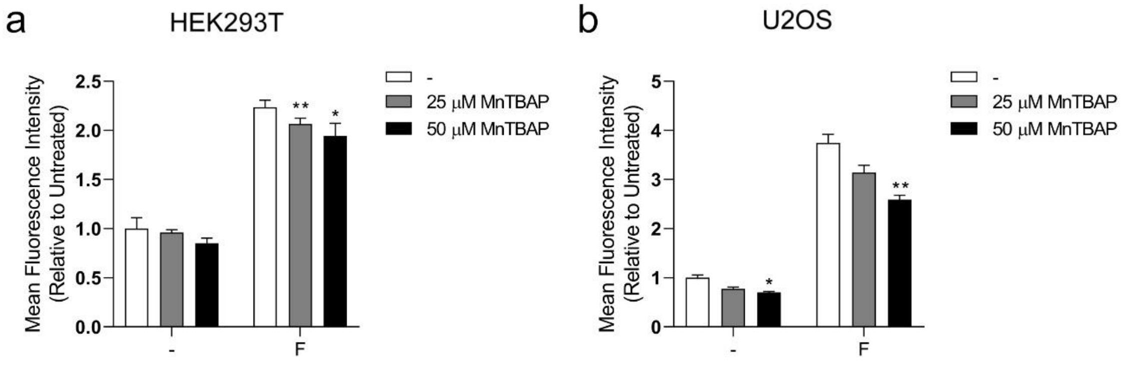

MnTBAP partially reduces fluorizoline-induced superoxide production. (**a**) HEK293T and (**b**) U2OS cells were pre-treated with the indicated doses of MnTBAP for 1 h and treated with 15 or 20 μM fluorizoline (F), respectively, for 4 h. Cells were loaded with 5 μM MitoSOX 15 min prior to the measurement. Superoxide production was measured by flow cytometry and is expressed as the mean fluorescence intensity relative to the untreated cells (-). * $p < 0.05$, ** $p < 0.01$, MnTBAP vs.-($n = 4$).

Figure S9

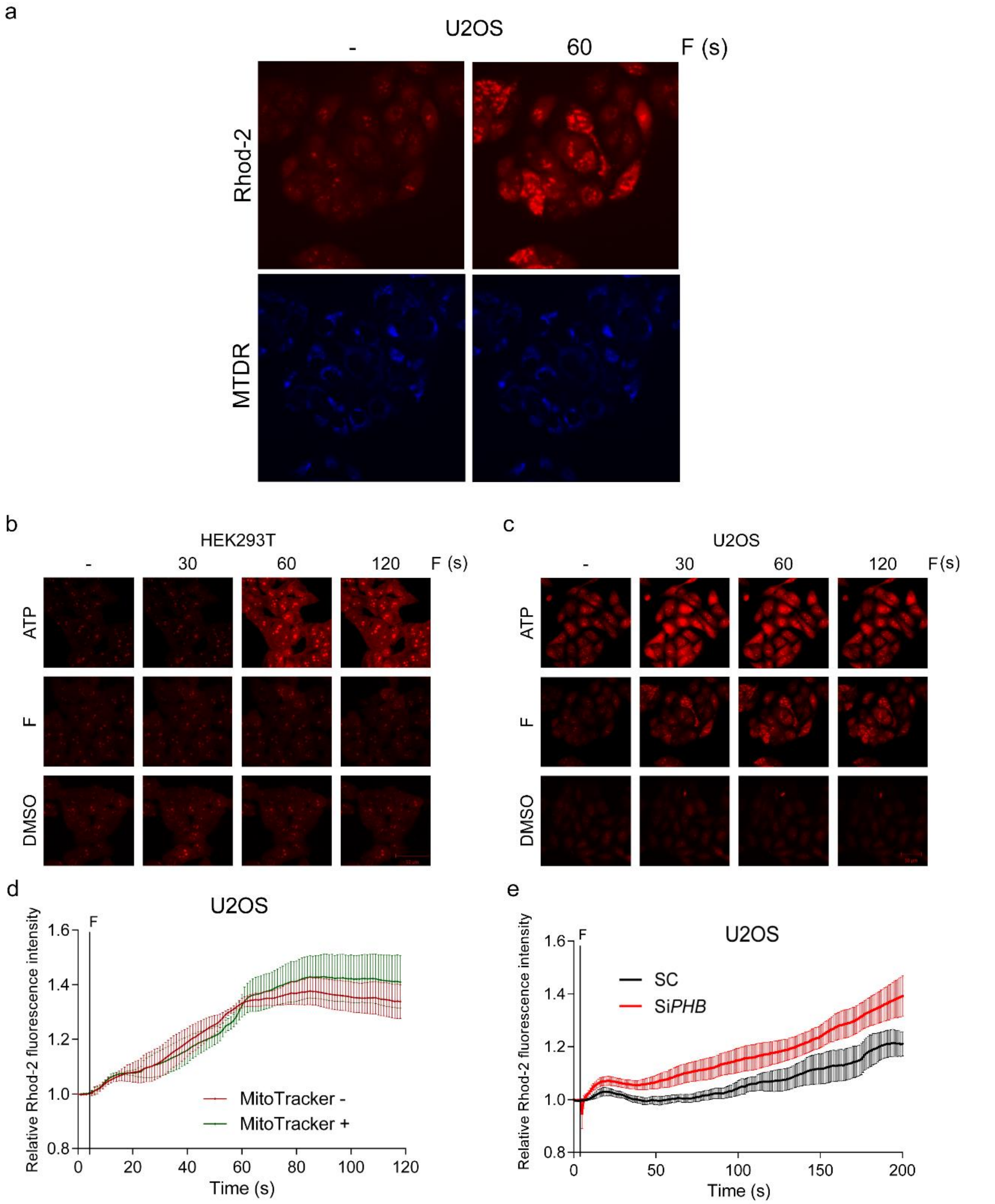

Fluorizoline increases cytosolic and mitochondrial calcium concentration in U2OS cells. (**a**,**c**–**e**) U2OS and (**b**) HEK293T cells were loaded with 5 μM Rhod-2 (Ex/Em of Ca^{2+}-bound form = 552/581 nm) 30 min prior to the start of the experiment and with 0.25 μM MitoTracker Deep Red 5 min prior to the start of the experiment. e U2OS cells were transfected with scramble (SC) or *PHB* siRNA (si*PHB*) for 72 h. Live-cell images were acquired at 1 s intervals for 2 min starting 5 s prior to treatment. HEK293 and U2OS cells were treated with 15 or 20 μM fluorizoline (F), respectively, with 100 μM ATP or with the corresponding volume of vehicle (DMSO). (**a**–**c**) These are representative images of at least three experiments. (**d**) Variations in Rhod-2 intensity were quantified in MitoTracker − and MitoTracker + pixels using ImageJ software.

Figure S10

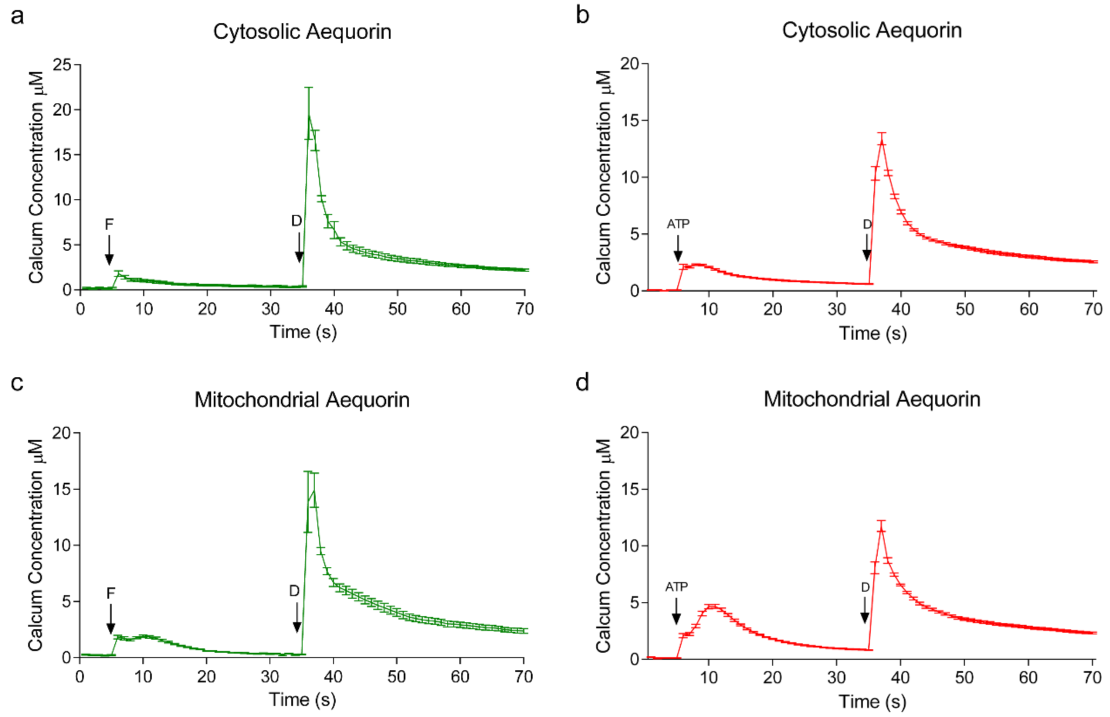

Fluorizoline increases cytosolic and mitochondrial calcium concentration in HEK293T cells. HEK293T cells were transfected with (**a**,**b**) cytosolic or (**c**,**d**) mitochondrially targeted aequorin for 48 h. After that, cells were pre-treated with 5 µM coelenterazine and were treated with either (**a**,**c**) 15 µM fluorizoline (F) or (**b**,**d**) 100 µM ATP as a positive control 5 s after the start of the acquisition. Luminescence was read in a plate reader at 1 s intervals. Cells were permeabilized 35 s after the start of the acquisition with 1 mM digitonin (D) in order to normalize between samples. Data represent the mean value for each second ± SEM (n = 3).

Figure S11

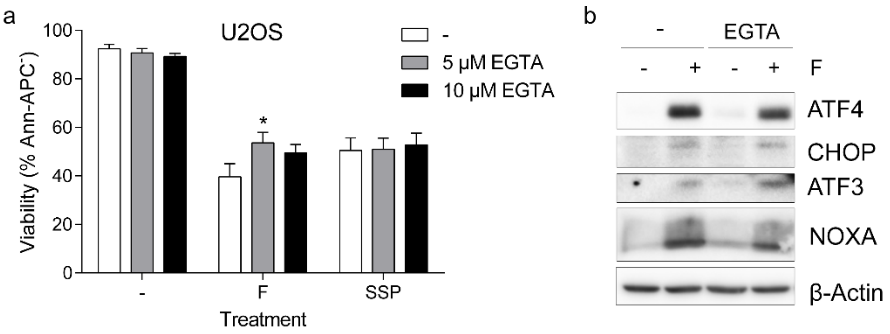

EGTA reduces fluorizoline-induced apoptosis in U2OS cells. (**a**,**b**) U2OS cells were pre-treated with the indicated doses or (**b**), with 10 µM EGTA for 1 h and then treated either with 20 µM fluorizoline or with 0.5 µM staurosporine (SSP) for (**b**) 4 h or (**a**) 24 h. (**a**) Viability was measured by flow cytometry, and it is expressed as the mean ± SEM of the percentage of non-apoptotic cells (annexin V-negative). * $p < 0.05$, EGTA vs.-(n = 3). (**b**) Protein levels were analyzed by Western blot. β-actin was used as a loading control. These are representative images of at least three independent experiments.
